# US Health Care Workforce Changes During the First and Second Years of the COVID-19 Pandemic

**DOI:** 10.1001/jamahealthforum.2021.5217

**Published:** 2022-02-25

**Authors:** Jonathan Cantor, Christopher Whaley, Kosali Simon, Thuy Nguyen

**Affiliations:** 1 RAND Corporation, Santa Monica, California; 2 O'Neill School of Public and Environmental Affairs, Indiana University, Bloomington, Indiana; 3 Department of Health Management and Policy, School of Public Health, University of Michigan, Ann Arbor

## Abstract

This article quantifies changes in employment and average wages of employees of 6 key health care organizations during the COVID-19 pandemic.

## Introduction

The COVID-19 pandemic has disrupted the US health care workforce owing to changes in the use and finances of health care clinician offices and institutions, increased health risks, burnout from increased patient burdens, and child care disruptions.^1,2,3,4^ While federal programs have provided financial assistance to hospitals and institutions,^1^ the net effect of these forces on health care employment levels and wages has not been examined. Understanding trends in employment levels by health care settings and locations is critical for planning and responding to public health crises.

## Methods

We used industry- and county-level data from the US Bureau of Labor Statistics Quarterly Census of Employment and Wages (QCEW, which covers more than 95% of US jobs).^5^ We conducted 2 analyses to quantify changes in employment and average wages of employees of 6 key health care organizations (offices of physicians, offices of dentists, home health care services, hospitals, skilled nursing facilities [SNFs], and combined other facilities) during 2020 and the first 6 months of 2021. First, we examined quarterly national trends in health care employment and average wages between March 31, 2020 (2020-Q1), and June 30, 2021 (2021-Q2), relative to 2019 (pre-COVID levels). Second, we examined associations between the 12-month changes in employment levels during 2019 to 2021, COVID-19 burden, and pre-COVID physician-to-population ratio. Multivariable linear regression models were conducted with Stata, version 17.0. The study was granted Not Regulated status by the University of Michigan Medical School Institutional Review Board (HUM00207016).

## Results

Health care employment levels declined suddenly, from 22.2 million in 2019 to 21.1 million, in 2020-Q2—a 5.2% decline (vs a 9.0% decline in all industries), and considerably rebounded to 21.8 million in 2021-Q2. Average wages within the health care sector increased at a lower rate relative to all industries’ changes (2020: 5.0% vs 6.7%; and 2021-Q2: 1.5% vs 6.9%).

Employment declines varied by types of health care organizations (Figure), with the largest declines in 2020-Q2 among offices of dentists (10.0%) and SNFs (8.4%). The smallest declines were among hospitals (2.5%) and offices of physicians (4.6%). While the employment level of most health care sectors rebounded to the pre-COVID levels in 2021-Q2, there were more declines in employment among SNFs (13.6% decline compared with 2019). Employees in SNFs exhibited the largest wage increases in 2020 (9.5%) and 2021 (6.3%), compared with 2019.

**Figure.**
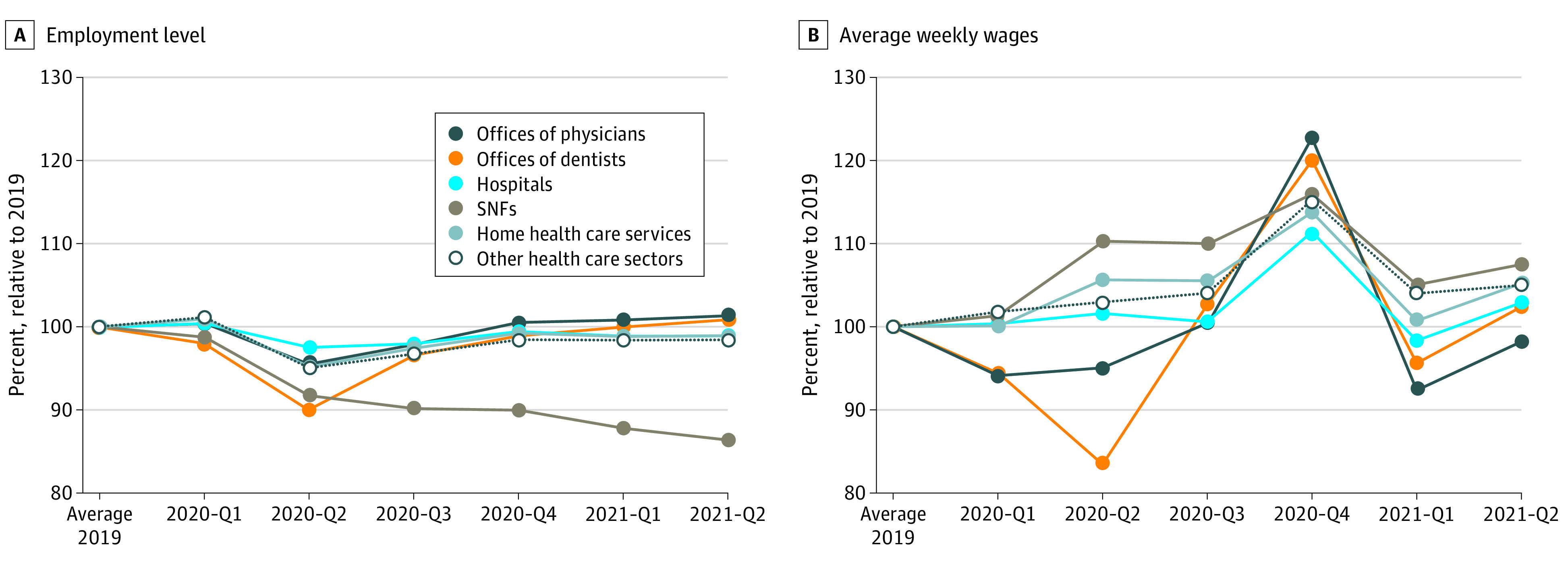
Trends of Quarterly Employment Level of the Health Care Sector During 2019-2021 Authors analyzed data from the Quarterly Census of Employment and Wages (QCEW) 2019-2021, provided by the US Bureau of Labor Statistics (BLS). Hospitals: all general and specialty hospitals (NAICS code: 622), offices of physicians (NAICS code: 6211), offices of dentists (NAICS code: 6212), home health care services (NAICS code: 6216), SNFs (NAICS code: 6231), and other health care sectors (outpatient care centers, 6214; other ambulatory health care services, 6219; medical and diagnostic laboratories, 6215; residential care, 6232; continuing care retirement communities, 6233; and other residential care, 6239). Abbreviations: NAICS, North American Industry Classification System; SNF, skilled nursing facility.

The Table highlights a statistically significant and positive association between the COVID-19 burden and 12-month percent change in employment levels among SNFs: the adjusted 2020 employment level in SNFs relative to 2019 was 105.2% among counties with the lowest quintile of COVID-19 cases and only 90.4% among counties with the top 20% burden (*P* = .008). Compared with the top 20%, counties with the lowest physician-to-population ratios tended to have higher adjusted employment levels in offices of physicians (107.8% vs 97.9%, *P* = .04) and among offices of dentists (110.1% vs 98.4%, *P* = .02). These associations were not statistically significant among other types of institutions.

**Table.** Associations Between Physician-to-Population Ratio, COVID-19 Burden, and Changes in Health Care Employment Level (12-Month Change During 2019-Q2 to 2020-Q2)^a^

Characteristic	Skilled nursing facilities	Offices		Home health care services	Hospitals
		Physicians	Dentists		
**COVID-19 burden (cases per 100 000 population)**					
Quintile 2 vs quintile 1 (highest)	8.17 (3.20 to 13.2)^b^	−3.27 (−8.38 to 1.84)	−1.86 (−6.59 to 2.87)	1.54 (−5.36 to 8.45)	60.3 (−57.3 to 177.9)
Quintile 3 vs quintile 1	6.12 (1.47 to 10.8)^c^	−1.19 (−6.08 to 3.70)	−1.68 (−6.09 to 2.73)	−5.12 (−14.0 to 3.78)	−11.9 (−46.1 to 22.4)
Quintile 4 vs quintile 1	15.2 (5.23 to 25.1)^b^	0.53 (−6.33 to 7.40)	−1.76 (−7.97 to 4.45)	−9.32 (−19.1 to 0.48)	−5.39 (−44.8 to 34.0)
Quintile 5 (lowest) vs quintile 1	14.9 (4.09 to 25.7)^b^	1.07 (−6.37 to 8.51)	−0.12 (−6.66 to 6.41)	−2.54 (−17.4 to 12.4)	10.3 (−27.9 to 48.6)
**MDs per 100 000 population**					
Quintile 2 vs quintile 1 (highest)	3.90 (−0.43 to 8.23)	1.36 (−1.93 to 4.65)	−1.73 (−5.13 to 1.67)	−4.54 (−12.7 to 3.58)	−33.5 (−101.9 to 35.0)
Quintile 3 vs quintile 1	1.56 (−6.80 to 9.91)	3.16 (−1.89 to 8.21)	1.41 (−3.20 to 6.01)	3.20 (−10.9 to 17.3)	−51.4 (−156.8 to 54.0)
Quintile 4 vs quintile 1	1.56 (−5.83 to 8.95)	8.98 (−0.98 to 18.9)	−0.081 (−7.01 to 6.85)	10.8 (−9.42 to 31.0)	−3.13 (−49.8 to 43.6)
Quintile 5 (lowest) vs quintile 1	−7.87 (−17.5 to 1.79)	9.87 (0.51 to 19.2)^c^	11.7 (2.03 to 21.3)^c^	−4.86 (−20.9 to 11.2)	−95.2 (−296.5 to 106.1)
Dependent variable					
Mean (SD)	98.13 (35.35)	101.20 (39.57)	98.95 (34.57)	106.54 (54.44)	117.99 (455.61)
Median	93.44	96.47	95.04	95.81	97.71
Observations (counties)^d^	1152	1980	1976	1222	548

Abbreviation: BLS, US Bureau of Labor Statistics; MD, Medical Doctor; Q2, second quarter; QCEW, Quarterly Census of Employment and Wages.

^a^ QCEW data between 2019-Q2 and 2020-Q2. The adjusted changes in outcomes are calculated from multivariable linear regressions of the 12-month percent change in employment on quintiles of COVID-19 cases per 100 000 residents and quintiles of active MDs per 100 000 residents. Unemployment rate, shares of racial minorities, shares of 65 years and older populations, share of rural population, adults’ insurance rate, median household income, and population, were included to account for pre-COVID demographic and socioeconomic predictors of employment changes. Standard errors were clusters in states. The 95% CIs are reported in parentheses.

^b^ *P* < .01.

^c^ *P* < .05.

^d^ These analyses exclude small counties where the BLS censors employment data to protect the identity of employers in counties with few establishments per industry.

## Discussion

Protecting the health care sector has been a priority in the pandemic.^6^ We documented changes in health care employment levels and wages during 2020 and 2021. We found substantial employment declines among SNFs, which were more severe in counties with high COVID-19 burden. The 2020 declines in employment among offices of physicians and offices of dentists were relatively smaller in counties with lower pre-pandemic physician-to-population ratios.

Limitations include the exclusion of certain counties with censored data and our inability to examine mechanisms for workforce changes. Future research is needed to understand if organizations are demanding fewer workers or fewer workers are willing to work at health care positions. Overall, our results imply that intensified early efforts are needed to protect the health care workforce in future pandemics.
